# Tumor Necrosis Factor Is a Therapeutic Target for Immunological Unbalance and Cardiac Abnormalities in Chronic Experimental Chagas' Heart Disease

**DOI:** 10.1155/2014/798078

**Published:** 2014-07-22

**Authors:** Isabela Resende Pereira, Glaucia Vilar-Pereira, Andrea Alice Silva, Otacilio Cruz Moreira, Constança Britto, Ellen Diana Marinho Sarmento, Joseli Lannes-Vieira

**Affiliations:** ^1^Laboratório de Biologia das Interações, Instituto Oswaldo Cruz/Fiocruz, Avenida Brasil 4365, 21045-900 Rio de Janeiro, RJ, Brazil; ^2^Departamento de Patologia, Universidade Federal Fluminense, 24033-900 Niterói, RJ, Brazil; ^3^Laboratório de Biologia Molecular e Doenças Endêmicas, IOC/Fiocruz, 21045-900 Rio de Janeiro, RJ, Brazil

## Abstract

*Background*. Chagas disease (CD) is characterized by parasite persistence and immunological unbalance favoring systemic inflammatory profile. Chronic chagasic cardiomyopathy, the main manifestation of CD, occurs in a TNF-enriched milieu and frequently progresses to heart failure. *Aim of the Study*. To challenge the hypothesis that TNF plays a key role in *Trypanosoma cruzi*-induced immune deregulation and cardiac abnormalities, we tested the effect of the anti-TNF antibody Infliximab in chronically *T. cruzi*-infected C57BL/6 mice, a model with immunological, electrical, and histopathological abnormalities resembling Chagas' heart disease. *Results*. Infliximab therapy did not reactivate parasite but reshaped the immune response as reduced TNF mRNA expression in the cardiac tissue and plasma TNF and IFN*γ* levels; diminished the frequency of IL-17A^+^ but increased IL-10^+^ CD4^+^ T-cells; reduced TNF^+^ but augmented IL-10^+^ Ly6C^+^ and F4/80^+^ cells. Further, anti-TNF therapy decreased cytotoxic activity but preserved IFN*γ*-producing VNHRFTLV-specific CD8^+^ T-cells in spleen and reduced the number of perforin^+^ cells infiltrating the myocardium. Importantly, Infliximab reduced the frequency of mice afflicted by arrhythmias and second degree atrioventricular blocks and decreased fibronectin deposition in the cardiac tissue. *Conclusions*. Our data support that TNF is a crucial player in the pathogenesis of Chagas' heart disease fueling immunological unbalance which contributes to cardiac abnormalities.

## 1. Introduction

Immunological unbalance with high levels of cytokines in the serum [1–5] is detected in patients with the cardiac form of Chagas disease (DC), a neglected tropical disease caused by the protozoan parasite* Trypanosoma cruzi* [6]. Importantly, increased plasma tumor necrosis factor (TNF) levels are related to the degree of left ventricular dysfunction in patients with chronic chagasic cardiomyopathy (CCC), the most frequent manifestation of CD [1, 3]. Chagas' heart disease is marked by persistence of low parasite load in the cardiac tissue and progressive fibrosis with remodeling of the myocardium and vasculature, which commonly progresses to heart failure and dilation [6]. In patients with CCC, the low-grade heart inflammation occurs in an inflammatory milieu enriched in the inflammatory cytokines TNF and interferon (IFN)*γ* [7]. Importantly, the number of TNF-producing cells in the cardiac tissue is associated with the presence of heart failure in CD patients [8]. In noninfectious conditions, the participation of TNF in ischemic and dilated heart disorders is supported by several observations, including elevated plasma TNF levels, and raised the proposal of using TNF blocking as immunotherapeutic strategy for improving the severity of heart diseases [9]. Antagonists of TNF as Etanercept (soluble dimeric human TNFR2/p75-IgG1 Fc fusion protein that binds to TNF and members of lymphotoxin family, neutralizing soluble TNF and LT*α*3 with similar potency) and Infliximab (chimeric monoclonal antibody with human IgG1*κ* Fc and murine variable regions that binds to both soluble and transmembrane TNF) have shown efficacy in a variety of immune-mediated inflammatory diseases [10, 11].

In experimental acute* T. cruzi* infection, the frequencies of TNF^+^ and TNF receptor 1/p55^+^ (TNFR1^+^) cells are increased [12]. Additionally, in acute* T. cruzi* infection TNFR1 signaling is crucial for parasite resistance [13] but also involved in heart tissue damage [12]. Moreover, the treatment of acutely* T. cruzi*-infected mice with the anti-TNF antibody Infliximab did not impact heart parasitism but reduced fibronectin (FN) deposition in the heart and ameliorated cardiomyocyte lesion in association with reduced CD8-enriched myocarditis [12]. These and other circumstantial findings led to the hypothesis that TNF signaling plays a role in the pathogenesis of heart tissue damage in* T. cruzi* infection [14]. This idea was previously challenged by administration of the soluble TNFR2 Etanercept to chronically infected hamsters with signs of CCC. This therapy did not alter blood and cardiac parasitism but significantly aggravated CCC in hamsters [15]. Interestingly, short treatment with Infliximab initiated three-month postinfection diminished cardiac TNF mRNA expression and CD8-enriched myocarditis in* T. cruzi*-infected rats, without evidence of parasitism reactivation but in presence of increased circulating IL-10 levels [16]. This regulatory cytokine was associated with the benign evolution of Chagas' heart disease [17].

Recently, we proposed that perforin (Pfn)^+^ and IFN*γ* ^+^ CD8^+^ T-cells infiltrating the cardiac tissue play antagonist roles in CCC [18].* In vitro* experiments support that Infliximab depletes a Pfn^+^CD8^+^ T-cell population which express TNF on cell surface [19]. More recently, in patients with a chronic inflammatory condition TNF neutralization was shown to downregulate IL-17 [20], a cytokine upregulated in cardiopathic CD patients [4]. Based on these data, we hypothesized that* in vivo* therapeutic intervention targeting TNF could selectively interfere with the nonbeneficial Pfn^+^CD8^+^ T-cells invading the cardiac tissue and also downregulate the Th17 profile associated with CCC. We, therefore, challenged the hypothesis that TNF fuels immunological unbalance which promotes Chagas' heart disease. For that, we used an experimental model of CCC occurring in parallel to high plasma TNF levels [18, 21] and short treatment with the monoclonal antibody Infliximab aiming to block TNF biological activities.

## 2. Materials and Methods

### 2.1. Ethical Information

Mice obtained from the animal facilities of the Oswaldo Cruz Foundation (CECAL/Fiocruz, Rio de Janeiro, Brazil) were housed under specific pathogen-free conditions in a 12-hour light-dark cycle with access to food and water* ad libitum*. Our protocols were approved by the Institutional Committee for Animal Ethics of Fiocruz (CEUA/Fiocruz, License 004/09). All presented data were obtained from three independent experiments (D2, T3, and T4, Experiment Register Books number 4 and number 5, LBI/IOC-Fiocruz).

### 2.2. Experimental Infection

Five- to seven-week-old female C57BL/6 (H-2^b^) mice were intraperitoneally infected with 100 blood trypomastigotes (bt) of the Colombian strain of* T. cruzi*, and parasitemia was employed as a parameter to establish acute and chronic phases [18]. Sex- and age-matched noninfected mice were kept in parallel. Each experimental group was composed of three to fifteen animals.

### 2.3. Anti-TNF Therapy

**Figure 1:**
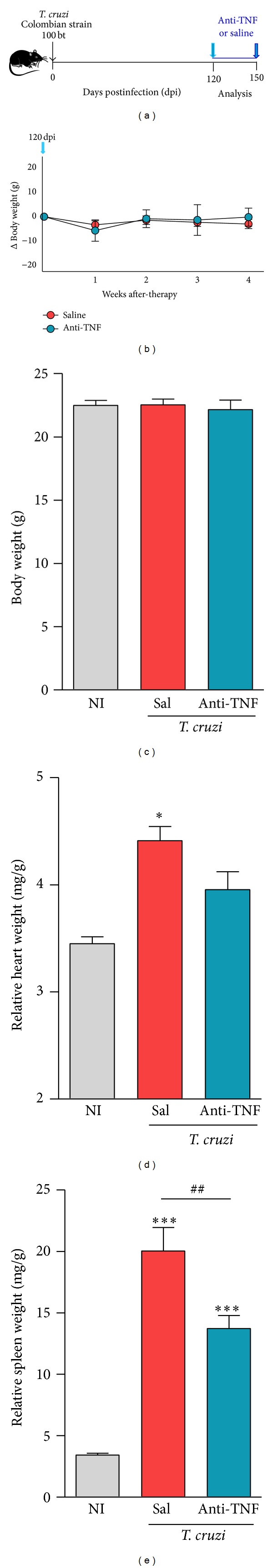
Anti-TNF therapy reduces* Trypanosoma cruzi*-induced splenomegaly in experimental model of chronic Chagas' heart disease. (a) C57BL/6 mice were infected with 100 bt of the Colombian* T. cruzi* strain and received saline or anti-TNF Infliximab 48-hour intervals from 120 (light blue arrow) to 150 days postinfection (dpi); noninfected mice received saline injections; all mice were analyzed at 150 (dark blue arrow) dpi. (b) Treatments were initiated at 120 dpi (blue arrow) and variation of body weight (g) was registered weekly. (c) Body weight (g), (d) relative heart weight (mg/g), and (e) relative spleen weight (mg/g) were analyzed at 150 dpi. *P < 0.05 and ***P < 0.001,* T. cruzi*-infected mice (saline injected or anti-TNF treated) compared to NI controls. ^##^ *P* < 0.01, anti-TNF-treated compared to saline-injected* T. cruzi*-infected mice. Bar represents the mean ± SD of the studied group (9 to 15 mice). Representative data from three independent experiments are shown.

C57BL/6 mice were given subcutaneous injections of 0.1 mL of apyrogenic saline (BioManguinhos, Fiocruz, Rio de Janeiro, RJ, Brazil) containing 10 *μ*g of anti-human TNF blocking antibody (Infliximab, Remicade), a gift from Schering-Plough of Brazil, 48-hour intervals from 120 to 150 days postinfection (dpi). Infliximab was previously shown to block* in vivo* TNF biological activities in murine and rat models [16, 22]. For injection control, sex- and age-matched noninfected mice received apyrogenic saline, according to our therapeutic schemes (Figure 1(a)). This group is, thereafter, referred to as noninfected (NI) controls.

### 2.4. Reagents and Antibodies

For functional assays, the H-2K^b^-restricted VNHRFTLV peptide from the amastigote surface protein 2 (ASP2) [18] was synthesized by GenScript USA Inc. (USA). For ELISpot anti-mouse IFN*γ* (clone R4-6A2) was used for capture, and biotin-conjugated anti-mouse IFN*γ* antibody (clone XMG1.2) and alkaline phosphatase-labeled streptavidin for detection were obtained from BD PharMingen (USA). For immunohistochemical staining (IHS) we use the polyclonal rabbit anti-mouse FN (Gibco-BRL, USA), anti-mouse F4/80 (CALTAG, USA), anti-mouse CD8a (53-6.7), and anti-mouse CD4 (clone GK1.5) supernatants were produced in our laboratory (LBI/IOC-Fiocruz, Brazil), biotinylated anti-rabbit immunoglobulin, biotinylated anti-rat immunoglobulin, and peroxidase-streptavidin complex were purchased form Amersham (UK). The monoclonal antibodies anti-mouse Pfn (clone CB5.4, Alexis Biochemicals, USA) and anti-IFN*γ* (clone R4-6A2, BD PharMingen, USA) produced in rat were also used in IHS. For flow cytometry studies, the reagents and antibodies recognizing mouse molecules purchased from BD Pharmingen (USA) were PE-Cy7-anti-TCR*α* *β* (clone H57-597), APC-anti-CD8a (clone 53-6.7), FITC-anti-CD4 (clone GK1.5), APC-anti-Ly6C (clone HK1.4), PE-Cy7-anti-TNF (clone MP6-XT22), PerCP-anti-CD4 (clone GK1.5), PE-anti-CD8a (clone 53-6.7), FITC-anti-IL-17A (clone eBio17B7), APC-anti-IL-10 (clone JES5-16E3), APC-streptavidin, FITC-anti-CD8a (clone 53-6.7), and PE-anti-TNF (clone MP6-XT22). The PE-Texas-red-anti-F4/80 (clone BM8) was made by Invitrogen (USA), the PE-anti-IL-10 (clone JES5-2A5) was made by CALTAG Laboratories, and PE-conjugated anti-TNFR1/p55/CD120a (clone 55R-286) was purchased from BioLegend (USA). Appropriate controls were prepared by replacing the antibodies with the corresponding serum, purified immunoglobulin, or fluorochrome-matched isotype. All antibodies and reagents were used according to the manufacturers' instructions.

### 2.5. Real-Time Quantitative RT-PCR for Detection of Cytokines mRNA

For real-time quantitative RT-PCR (qRT-PCR), the hearts were harvested, washed to remove blood clots, weighed, and frozen in RNAlater (number AM7021, Life Technologies, USA). The total RNA was extracted from the same sample used for DNA extraction for parasite detection using TRI-Reagent (Sigma-Aldrich, USA). All reverse transcriptase reactions were performed using a SuperScript III Kit (number 18080-051) and qRT-PCR was performed using TaqMan gene expression assays for the cytokine TNF (number Mm00443258_m1) and the endogenous housekeeping control genes glyceraldehyde 3-phosphate dehydrogenase (GAPDH) (number Mm99999915_g1) and HPRT (number Mm01545399_m1), purchased from Life Technologies (USA). The reactions were performed and analyzed as previously described [21].

### 2.6. Real-Time Quantitative PCR for Parasite Detection

Hearts were collected and processed as described in Section 2.5. Total DNA was extracted from heart samples using TRI-Reagent (Sigma-Aldrich, USA). The purified DNA was analyzed by real-time quantitative PCR (qPCR) using primers Cruzi 1 (5′-AST CGG CTG ATC GTT TTC GA-3′) and Cruzi 2 (5′-AAT TCC TCC AAG CAG CGG ATA-3′) for* T. cruzi* nuclear satellite DNA and GAPDH gene (GAPDH Fw 5′-CCA CTC ACG GCA AAT TCA ACG GC-3′ and GAPDH Rv 5′-CCA CCC TTC AAG TGG GCC CCG-3′), as an internal control from the mouse DNA. Parasitic load quantification was obtained by absolute quantification of* T. cruzi* DNA, following normalization by heart weight. The standard curve for the absolute quantification was generated by a 1 : 10 serial dilution of DNA extracted from the Colombian strain epimastigote culture stocks, ranging from 10^6^ to 10 parasite equivalents. All procedures and analyses were performed as previously described [23].

### 2.7. Detection of Cytokines in the Serum

A mouse cytometric bead array (CBA; Inflammation Kit (number 560485, Becton & Dickinson, USA)) was used to quantify cytokines in the serum according to the manufacturer's instructions. The fluorescence produced by the CBA beads was measured with a FACSCalibur instrument (Becton Dickinson, USA) and analyzed using FCAP Array software. Standard curves (1 pg/mL to 100 ng/mL) were generated in parallel. This method consistently detected concentrations above 10 pg/mL.

### 2.8. Nitric Oxide Quantification

Nitrate and nitrite (NO_*x*_) were determined in serum samples from NI and infected mice using Griess reagent and vanadium chloride III with a standard curve of 0.8–100 *μ*M NaNO_2_ and NaNO_3_ [23].

### 2.9. Flow Cytometry Analysis

Spleens were minced, and the red blood cells were removed using lysis buffer (Sigma, USA). The splenocytes were labeled, 100,000 to 300,000 events were acquired with a Beckman Coulter CyAn 7 Color flow cytometer (USA), and the data were analyzed with the Summit v.4.3 Build 2445 program (Dako, USA), as described elsewhere [18].

### 2.10. IFN*γ* Enzyme-Linked Immunospot (ELISpot) Assay

The ELISpot assay for the enumeration of IFN*γ*-producing cells was performed in triplicate as previously described [18]. Briefly, plates were coated with the capture anti-mouse IFN*γ* antibody (clone R4-6A2). Antigen-presenting cells were primed with the immunodominant H-2K^b^-restricted VNHRFTLV amastigote surface protein 2 (ASP2) peptide, incubated with freshly isolated splenocytes from experimental mice (5 × 10^5^ cells/well), incubated for 20 hours at 37°C and 5% CO_2_, and cytokine was detected using biotin-conjugated anti-mouse and revealed as previously described alkaline phosphatase-labeled streptavidin in presence of a solution of NBT and BCIP (Sigma, USA). The mean number of spots in triplicate wells was determined for each experimental condition and the number of specific IFN*γ*-secreting T-cells was calculated by estimating the stimulated spot count/10^6^ cells using a CTL OHImmunoSpot A3 Analyzer (USA), as previously described [18].

### 2.11. *In Vivo* Cytotoxicity Assay

For the* in vivo* cytotoxicity assays, splenocytes from naïve C57BL/6 mice were divided into two populations and labeled with the fluorogenic dye CFSE (Molecular Probes, USA) at a final concentration of 10 *μ*M (CFSE^high^) or 0.1 *μ*M (CFSE^low^). CFSE^high^ cells were coated with 2.5 *μ*M of the VNHRFTLV ASP2 peptide for 40 minutes at 37°C. The CFSE^low^ cells remained uncoated. Subsequently, the CFSE^high^ cells were washed and mixed with equal numbers of CFSE^low^ cells before intravenous injection (1-2 × 10^7^ cells per mouse) into C57BL/6 recipients sedated with ketamine and xylazine (100 mg/kg and 5 mg/kg, resp.). Spleen cells were collected from the recipient mice at 20 hours after adoptive cell transfer and fixed using 1.0% paraformaldehyde. All samples were acquired using a Beckman Coulter CyAn 7 Color flow cytometer (USA) and analyzed using the Summit v.4.3 Build 2445 program (Dako, Denmark) and the percentage of specific lysis was determined as previously described [18].

### 2.12. Immunohistochemistry

The animals were euthanized under anesthesia at 150 dpi and the hearts were removed, embedded in the tissue-freezing medium Tissue-Tek (Miles Laboratories, USA), and stored in liquid nitrogen. Three sections were analyzed per heart tissue. The phenotypes of the inflammatory cells colonizing the heart tissue and the FN deposition were characterized and analyzed as previously described [18, 24]. The numbers of CD4^+^, CD8^+^, F4/80^+^ (macrophages), IFN*γ* ^+^, and Pfn^+^ inflammatory cells were counted in 100 microscopic fields (400X magnification) per section. The FN-positive areas in 25 fields (12.5 mm^2^) per section were evaluated with a digital morphometric apparatus. The images were digitized using a color view XS digital video camera adapted to a Zeiss microscope and analyzed with AnalySIS AUTO Software (Soft Imaging System, USA). The data are presented as the percent of FN positive area in the cardiac tissue.

### 2.13. Electrocardiogram (ECG) Registers

Mice were tranquilized with diazepam (10 mg/kg) and transducers were placed subcutaneously (DII). The traces were recorded for 2 min using a digital power lab 2/20 system connected to a bioamplifier at 2 mV for 1 second (PanLab Instruments, Spain). The filters were standardized between 0.1 and 100 Hz and the traces were analyzed using Scope software for Windows V3.6.10 (PanLab Instruments, Spain). The ECG parameters were analyzed as previously described [18].

### 2.14. Statistical Analysis

The data are expressed as arithmetic means ± standard deviation. Student's *t*-tests, ANOVA, or other appropriate tests were used to analyze the statistical significance of the observed differences. The Kaplan-Meier method was used to compare the survival times of the studied groups. All statistical tests were performed with GraphPad Prism. Differences were considered statistically significant when *P* < 0.05.

## 3. Results

### 3.1. Anti-TNF Therapy Does Not Affect Body Weight but Reduced the* T. cruzi*-Induced Splenomegaly

To test the hypothesis that TNF is a hub in the immunological and cardiac abnormalities, therapy with Infliximab was initiated at 120 dpi; when high TNF levels in the serum [21], splenomegaly and signs of CCC as cardiomegaly and electrical abnormalities [18] are detected. We used a therapeutic scheme with 48-hour interval injections; all parameters were analyzed at 150 dpi (Figure 1(a)), when all saline-injected and anti-TNF-treated* T. cruzi*-infected C57BL/6 mice were alive (data not shown). Infliximab and saline administration did not affect the body temperature (data not shown) and weight of* T. cruzi*-infected C57BL/6 mice (Figure 1(b)). Indeed, at 150 dpi, there was no difference in body weight when* T. cruzi*-infected mice (injected with saline or treated with anti-TNF) were compared to age-matched NI controls (Figure 1(c)). Short Infliximab therapy significantly decreased (3.95 ± 0.33 mg/g; *P* < 0.01) the cardiomegaly detected in not-treated (4.8 ± 0.38 mg/g) chronically infected mice. However, there was no difference when Infliximab-treated mice were compared with saline-injected (4.31 ± 0.66 mg/g; *P* = 0.156) chronically infected mice (Figure 1(d)). At 120 dpi, a remarkable splenomegaly due to increased number of splenocytes is noticed in Colombian-infected C57BL/6 mice compared to noninfected mice [18]. At 150 dpi, splenomegaly was also detected in saline-injected mice. However, anti-TNF therapy significantly reduced the number of total splenocytes (data not shown) and splenomegaly (Figure 1(e)).

### 3.2. Anti-TNF Therapy Does Not Reactivate* T. cruzi* Infection

**Figure 2:**
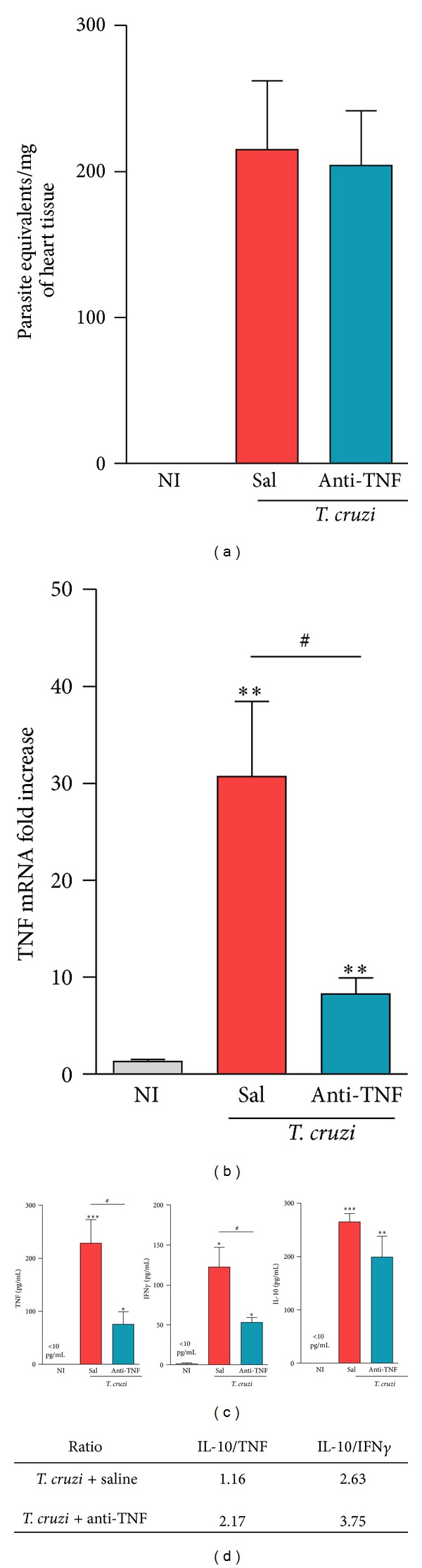
Therapy with Infliximab does not reactivate* Trypanosoma cruzi* but reduces TNF mRNA expression in the heart tissue and inflammatory cytokine profile in the serum of chronically infected mice. C57BL/6 mice infected with 100 bt of the Colombian* T. cruzi* strain were treated from 120 to 150 dpi and analyzed at 150 dpi. (a) Detection of parasite DNA (equivalents/mg) in the cardiac tissue. (b) Expression of TNF mRNA (fold increase) in the heart tissue. (c) Measure of cytokine concentrations in the serum. (d) Relative ratios of IL-10/TNF and IL-10/IFN*γ* levels in the serum in saline-injected and anti-TNF treated infected mice. *P < 0.05, **P < 0.01, and ***P < 0.001,* T. cruzi*-infected mice (saline injected or anti-TNF treated) compared to NI controls. ^#^ *P* < 0.05, anti-TNF-treated compared to saline-injected* T. cruzi*-infected mice. Bar represents the mean ± SD of the studied group (3 to 5 mice). These data represent three independent experiments.

At 150 dpi, heart tissue parasitism, detected by qPCR for genomic parasite DNA, persisted low in the chronic phase of* T. cruzi* infection of C57BL/6 mice (Figure 2(a)). Importantly, anti-TNF therapy with Infliximab did not reactivate* T. cruzi* infection in the central nervous tissue systemically (data not shown) and in the cardiac tissue of chronically infected mice (Figure 2(a)).

### 3.3. Treatment with Infliximab Reduces TNF mRNA Expression in the Heart Tissue

At 150 dpi, the expression of TNF mRNA was upregulated in the cardiac tissue of* T. cruzi*-infected mice compared to age-matched NI controls (Figure 2(b)). Notably, this TNF mRNA overexpression was significantly diminished after short anti-TNF therapy (Figure 2(b)).

### 3.4. TNF Neutralization Decreases the Levels of Inflammatory Cytokines but Preserves IL-10 Levels in the Serum

Chronic chagasic patients with severe myocardial involvement present a systemic inflammatory profile revealed as increased levels of cytokines (TNF, IFN*γ*, IL-10, IL-17, and IL-6) and the inflammatory mediator NO_*x*_ in the serum [4]. These features were reproduced in the experimental model of CCC in Colombian-infected C57BL/6 mice in comparison with age-matched NI controls (Figure 2(c) and Figures S1(a) and S1(b) available online at http://dx.doi.org/10.1155/2014/798078). Anti-TNF therapy tended to decrease the levels of IL-17A, IL-6, and NO_*x*_ in the serum (Figures S1(a) and S1(b)). Importantly, administration of Infliximab from 120 to 150 dpi significantly reduced the levels of TNF and IFN*γ* but did not influence the levels of IL-10 in the serum (Figure 2(c)). Consequently, anti-TNF therapy increased the IL-10/TNF and IL-10/IFN*γ* ratios in chronically infected mice (Figure 2(d)).

### 3.5. TNF Blocking Reduces the Accumulation of CD8^+^ Cells in the Heart Tissue

At 120 dpi, chronically Colombian-infected C57BL/6 mice show CD8-enriched myocarditis [18]. Anti-TNF therapy did not diminish the general inflammatory foci composed of CD8^+^, CD4^+^, and F4/80^+^ (macrophages) (Figure S2(a)) but reduced the number of CD8^+^ cells infiltrating the cardiac tissue (Figures S2(b) and S2(c)). Therefore, we tested whether it was a site restricted effect examining the cell composition of a secondary lymphoid tissue. The frequencies of CD4^+^ and CD8^+^ T-cells in the spleen persisted unchanged after anti-TNF administration (Figure S2(d)).

### 3.6. TNF Neutralization with Infliximab Reduces TNFR1 Expression on CD4^+^ and CD8^+^ T-Cells

**Figure 3:**
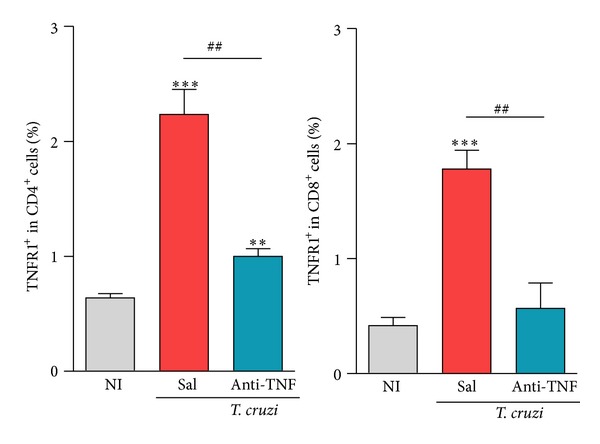
Anti-TNF administration reduces the frequency of TNFR1-expressing CD4^+^ and CD8^+^ T-cells in chronically* Trypanosoma cruzi*-infected mice. C57BL/6 mice infected with 100 bt of the Colombian* T. cruzi* strain were treated from 120 to 150 dpi and analyzed at 150 dpi. Frequency of TNFR1^+^ cells among CD4^+^ and CD8^+^ T-cells in the spleen [R1 (SSCxFSC) gated]. **P < 0.01 and ***P < 0.001,* T. cruzi*-infected mice (saline injected or anti-TNF treated) compared to NI controls. ^##^ *P* < 0.01, anti-TNF-treated compared to saline-injected* T. cruzi*-infected mice. Bar represents the mean ± SD of the studied group (4 to 6 mice). These data represent two independent experiments.

The frequencies of TNF^+^ and TNFR1^+^ splenic cells are upregulated in acute* T. cruzi* infection [12]. The frequencies of TNF-expressing CD4^+^ and CD8^+^ T-cells, which were also upregulated in chronically* T. cruzi*-infected mice, were not significantly modified by short therapy with anti-TNF (data not shown). The frequencies of CD4^+^TNFR1^+^ and CD8^+^TNFR1^+^ splenic T-cells were also markedly increased in chronically infected C57BL/6 mice compared to NI controls (Figure 3). Interestingly, anti-TNF therapy significantly decreased the frequency of TNFR1-bearing CD4^+^ and CD8^+^ T-cells compared with saline injection (Figure 3).

### 3.7. Anti-TNF Decreases TNF While Favors IL-10 Expression by Ly6C^+^ and F4/80^+^ Cells

**Figure 4:**
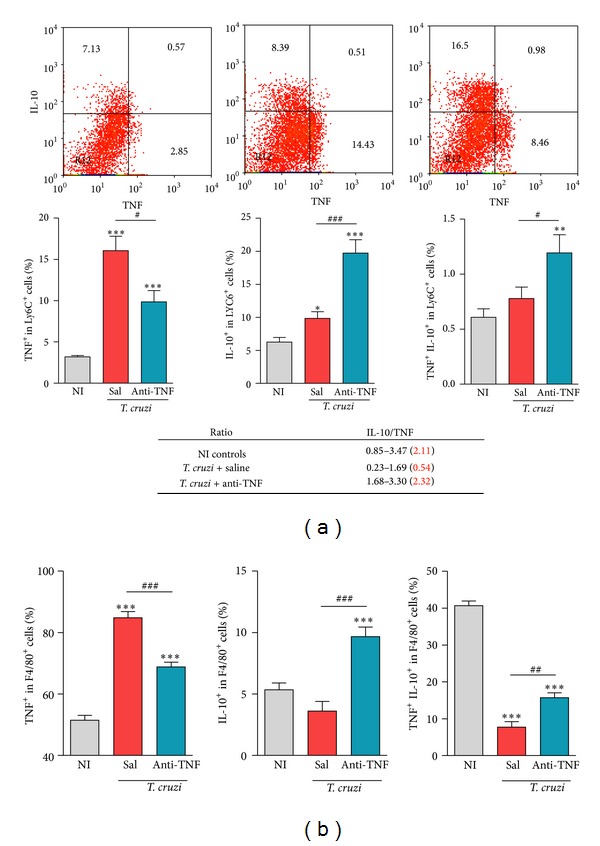
Anti-TNF therapy reduces the frequency of TNF^+^ cells but increases the frequency of IL-10^+^ and IL-10^+^TNF^+^ cells among Ly6C^+^ and F4/80^+^ macrophages in chronically* Trypanosoma cruzi*-infected mice. C57BL/6 mice infected with 100 bt of the Colombian* T. cruzi* strain were treated from 120 to 150 dpi and analyzed at 150 dpi. (a) Representative dot plots, frequency of TNF^+^, IL-10^+^, and TNF^+^ IL-10^+^ cells among Ly6C^+^ in the spleen [R1 (SSCxFSC) mononuclear cells gated], and ranges (means) of the IL-10/TNF relative ratios are shown. (b) Frequency of TNF^+^, IL-10^+^, and TNF^+^ IL-10^+^ double-positive cells among F4/80^+^ in the spleen [R1 (SSCxFSC) mononuclear cells gated]. *P < 0.05, **P < 0.01, and ***P < 0.001,* T. cruzi*-infected mice (saline injected or anti-TNF treated) compared to NI controls. ^#^ *P* < 0.05, ^##^ *P* < 0.01, and ^###^ *P* < 0.001, anti-TNF-treated compared to saline-injected* T. cruzi*-infected mice. Bar represents the mean ± SD of the studied group (4 to 6 mice). These data represent two independent experiments.

Monocytes from cardiopathic CD patients seem to be committed to high TNF production, while monocytes from patients with the indeterminate (IND) form of CD display modulatory profile with high IL-10 production [17]. Therefore, we tested the effect of anti-TNF therapy on inflammatory (TNF) and regulatory (IL-10) profiles of Ly6C^+^ and F4/80^+^ splenic cells in chronic* T. cruzi* infection. In comparison with NI controls, chronically saline-injected* T. cruzi*-infected mice presented a remarkable increase in the frequency of TNF^+^ cells among Ly6C^+^ (Figure 4(a)) and F4/80^+^ (Figure 4(b)) in R1(SSCxFSC) gated mononuclear splenocytes. Anti-TNF therapy significantly reduced the frequency of TNF-producing Ly6C^+^ (Figure 4(a)) and F4/80^+^ (Figure 4(b)) cells. Further, treatment with Infliximab upregulated the frequencies of IL-10^+^ single-positive and TNF^+^IL-10^+^ double-positive cells among Ly6C^+^ (Figure 4(a)) and F4/80^+^ (Figure 4(b)) cells. Interestingly, short anti-TNF therapy reestablished the IL-10/TNF ratio among Ly6C^+^ mononuclear splenocytes (means: 2.11 in NI controls; 0.54 in saline-injected versus 2.32 in anti-TNF-treated infected mice; Figure 4(a)).

### 3.8. TNF Neutralizing Antibody Diminishes the Frequency of IL-17A^+^ but Increased IL-10^+^ CD4^+^ T-Cells

**Figure 5:**
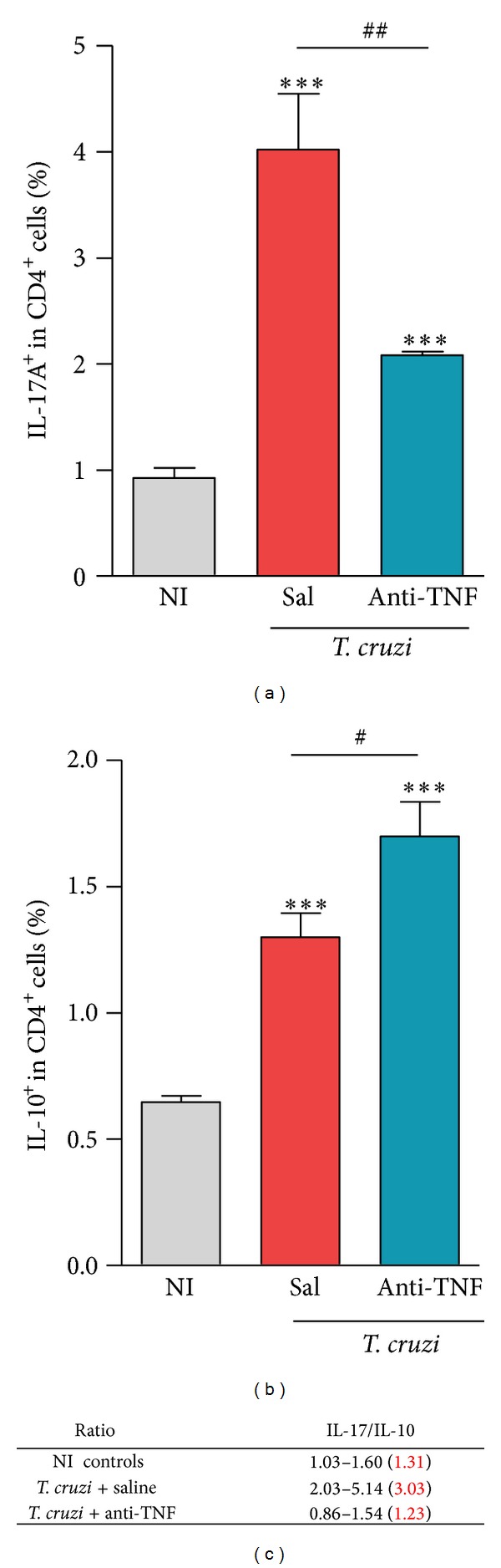
Anti-TNF therapy reduces the frequency of IL-17A^+^ CD4^+^ cells but increases the frequency of IL-10^+^ CD4^+^ cells in chronically* Trypanosoma cruzi*-infected mice. C57BL/6 mice infected with 100 bt of the Colombian* T. cruzi* strain were treated from 120 to 150 dpi and analyzed at 150 dpi. (a) Frequencies of IL-17A^+^ and (b) IL-10^+^ cells among CD4^+^ cells in the spleen [R1 (SSCxFSC)/R2 (TCR*α* *β*/CD4) gated]. (c) Relative ratios of IL-17A/IL-10 in saline-injected and anti-TNF treated infected mice. ***P < 0.001,* T. cruzi*-infected mice (saline injected or anti-TNF treated) compared to NI controls. ^#^ *P* < 0.05 and ^##^ *P* < 0.01, anti-TNF-treated compared to saline-injected* T. cruzi*-infected mice. Bar represents the mean ± SD of the studied group (4 to 6 mice). These data represent three independent experiments.

Increased frequency of IL-17A^+^ CD4^+^ cells was noticed in chronically infected mice compared to age-matched NI controls (Figure 5(a)). Recently, anti-TNF therapy was shown to downregulate IL-17 expression and the frequency of Th17 cells in ankylosing spondylitis patients [20]; therefore, we tested whether it was also the case in chronic* T. cruzi* infection. Interestingly, Infliximab administration reduced the frequency of IL-17A^+^ cells (Figure 5(a)). Conversely, anti-TNF therapy significantly enhanced the frequency of IL-10^+^ CD4^+^ cells, which was already upregulated during the chronic infection (Figure 5(b)). Interestingly, short anti-TNF therapy reestablished the IL-17A/IL-10 ratio among CD4^+^ T splenocytes (means: 1.31 in NI controls; 3.03 in saline-injected versus 1.23 in anti-TNF-treated infected mice; Figure 5(c)).

### 3.9. TNF Neutralization Reduces Cytotoxic Activity but Preserves IFN*γ*-Producing Parasite-Specific CD8^+^ T-Cells

**Figure 6:**
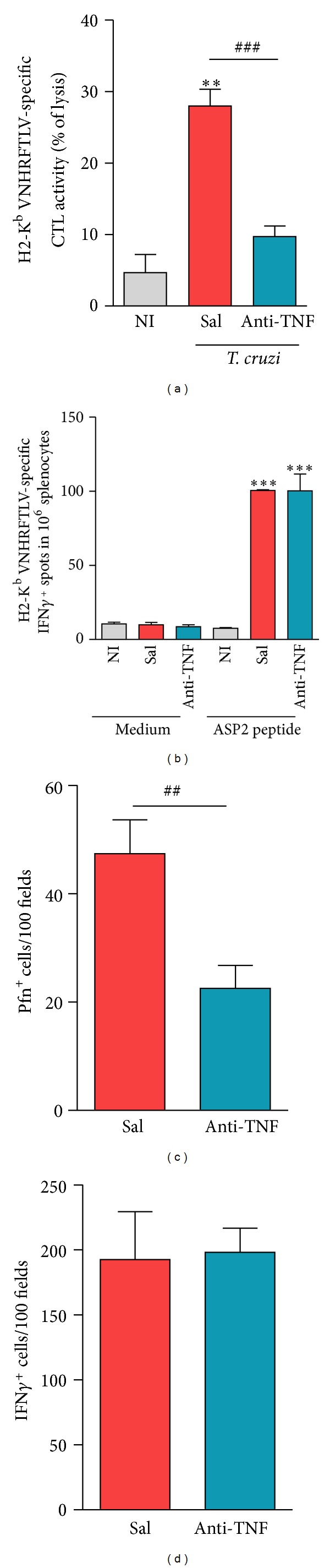
Anti-TNF therapy reshapes* Trypanosoma cruzi*-specific CTL activity and IFN*γ*-producing cells in spleen and the composition of Pfn^+^ and IFN*γ* ^+^ cells invading the cardiac tissue in chronically* Trypanosoma cruzi*-infected mice. C57BL/6 mice infected with 100 bt of the Colombian* T. cruzi* strain were treated from 120 to 150 dpi and analyzed at 150 dpi. (a) Frequency of* in vivo* specific lysis of H-2K^b^-restricted VNHRFTLV ASP2 peptide-labeled target cells in spleen. (b) Numbers of H-2K^b^-restricted VNHRFTLV ASP2 peptide IFN*γ* ^+^ cells (spots) among splenocytes. Immunohistochemical staining of (c) Pfn^+^ and (d) IFN*γ* ^+^ cells infiltrating the cardiac tissue, at 150 dpi. **P < 0.01 and ***P < 0.001,* T. cruzi*-infected mice (saline injected or anti-TNF treated) compared to NI controls. ^##^ *P* < 0.01 and ^###^ *P* < 0.001, anti-TNF-treated compared to saline-injected* T. cruzi*-infected mice. Bar represents the mean ± SD of the studied group (3 to 6 mice). These data represent three independent experiments.

Recently, we proposed that Pfn^+^ and IFN*γ* ^+^ CD8^+^ T-cells play antagonist roles in CCC [18]. Further,* in vitro* stimulus of CD8^+^ T-cells with* Mycobacterium* lysed in the presence of anti-TNF antibody reduced the effector cytotoxic activity [19]. Therefore, we hypothesized that* in vivo* therapeutic intervention targeting TNF could selectively interfere with distinct CD8^+^ T-cells modulating CD8^+^Pfn^+^ and cytotoxic CD8^+^ T-cell effectors (CTL) in chronic* T. cruzi* infection. Herein, we challenged this hypothesis. When compared to age-matched NI controls, there was a significant increase in* in vivo* CTL activity (Figure 6(a)) and IFN*γ*-producing (Figure 6(b)) CD8^+^ T-cells which recognize the immunodominant H-2K^b^-restricted VNHRFTLV ASP2 peptide in chronically infected mice, at 150 dpi. More importantly, short anti-TNF therapy reduced CTL activity (Figure 6(a)) but preserved IFN*γ*-producing (Figure 6(b)) CD8^+^ T-cells specific for the immunodominant VNHRFTLV ASP2 peptide.

### 3.10. Infliximab Administration Reduces the Number of Pfn^+^ Cells Infiltrating the Cardiac Tissue

We, then, examined whether anti-TNF therapy influenced the balance of cytotoxic (Pfn^+^) and inflammatory (IFN*γ* ^+^) cells composing the chronic* T. cruzi*-induced myocarditis. Compared to saline-injected mice, anti-TNF-treated mice showed reduced number of Pfn^+^ cells (Figure 6(c)) but similar number of IFN*γ* ^+^ cells (Figure 6(d)) infiltrating the cardiac tissue, supporting that Infliximab remolded chronic* T. cruzi*-induced myocarditis.

### 3.11. Anti-TNF Treatment Improves Chronic ECG Abnormalities

**Figure 7:**
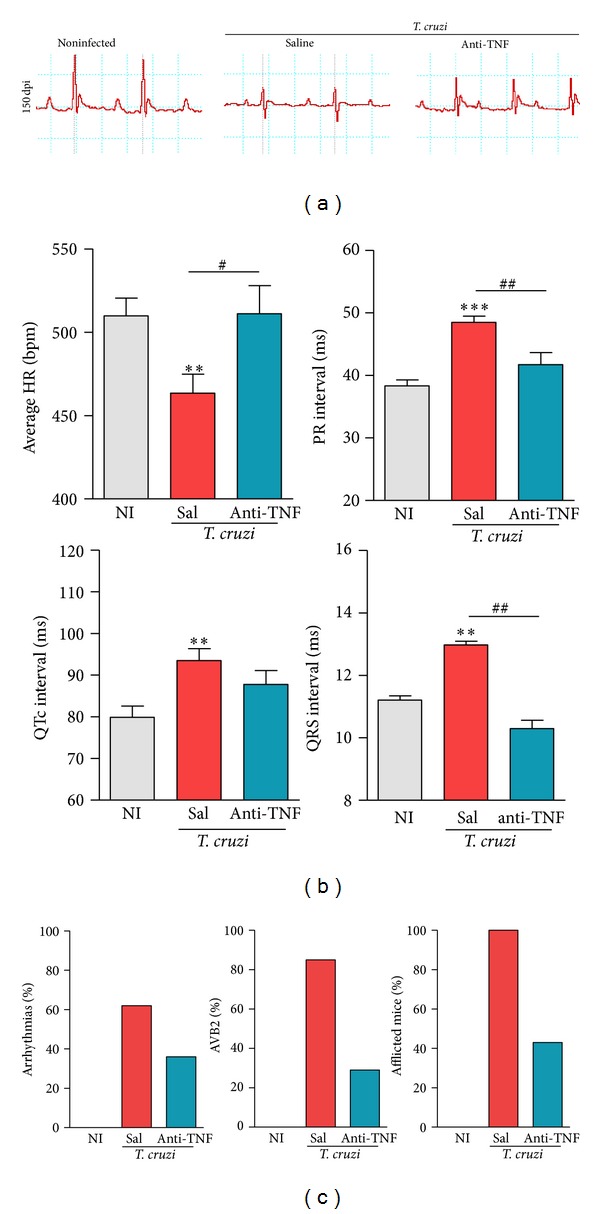
Anti-TNF therapy ameliorates electrical abnormalities in chronically* Trypanosoma cruzi*-infected mice. C57BL/6 mice infected with 100 bt of the Colombian* T. cruzi* strain were treated from 120 to 150 dpi and analyzed at 150 dpi. (a) Representative ECG register segments of sex- and age-matched NI controls and* T. cruzi*-infected mice injected with saline or anti-TNF, at 150 dpi. (b) Group data for the ECG records showing the heart rate (beats per minute, bpm), variation in the PR, QTc, and QRS intervals. (c) Summary of the group data from NI and* T. cruzi*-infected mice (injected with saline or anti-TNF) showing the frequency of mice presenting ART, AVB2 and afflicted by any ECG alterations. **P < 0.01 and ***P < 0.001,* T. cruzi*-infected mice (saline injected or anti-TNF treated) compared to NI controls. ^#^ *P* < 0.05 and ^##^ *P* < 0.01, anti-TNF-treated compared to saline-injected* T. cruzi*-infected mice. Bar represents the mean ± SD of the studied group (10 to 15 mice). These data represent three independent experiments.

Based on our results that anti-TNF downmodulates the expression of TNF and CTL activity, immune effectors hypothesized to be protagonist players of CCC pathogenesis [14, 18], we examined the effects of Infliximab in chronically* T. cruzi*-induced electrical abnormalities. At 120 dpi, when treatment with Infliximab was initiated, electrical abnormalities were already established in Colombian-infected C57BL/6 mice [18]. At 150 dpi, chronically infected C57BL/6 mice not-treated (data not shown) or injected with saline presented abnormalities in the electrical conduction system (Figure 7(a)), including low heart rate and prolonged PR, corrected QT (QTc) and QRS intervals (Figure 7(b)). Further, compared to NI controls, chronically infected mice showed a high proportion of mice afflicted by arrhythmias (ART), second-degree atrioventricular block (AVB2), and other ECG abnormalities (Figure 7(c)), including a low frequency of mice showing first-degree atrioventricular block (AVB1) and rare cases of fibrillation (data not shown). Importantly, anti-TNF therapy restored the normal heart rate, P wave duration (data not shown), and PR, QTc, and QRS intervals (Figure 7(b)). Notably, compared with their counterparts anti-TNF therapy reduced the proportions of mice afflicted by ART, AVB2, and any ECG abnormalities (Figure 7(c)).

### 3.12. Anti-TNF Treatment Ameliorates Chronic* T. cruzi*-Induced Heart Tissue Damage

**Figure 8:**
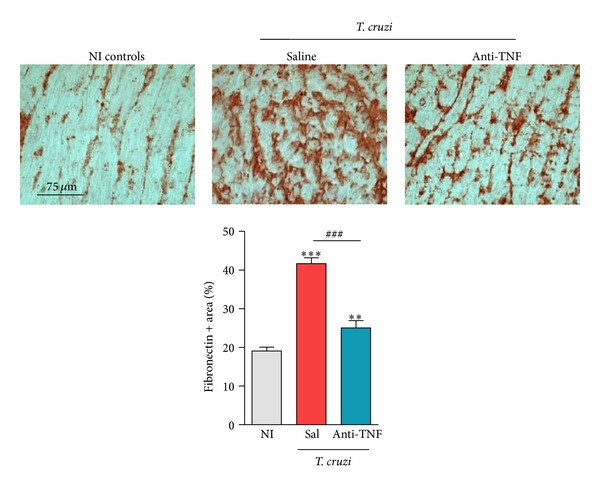
Anti-TNF therapy decreases fibronectin expression in the cardiac tissue of chronically* Trypanosoma cruzi*-infected mice. C57BL/6 mice infected with 100 bt of the Colombian* T. cruzi* strain were treated from 120 to 150 dpi and analyzed at 150 dpi. Representative heart sections from each experimental group analyzed by immunohistochemical staining to detect FN are shown. Data are expressed as percentage of FN-stained area (%).**P < 0.01 and ***P < 0.001,* T. cruzi*-infected mice (saline injected or anti-TNF treated) compared to NI controls. ^###^ *P* < 0.001, anti-TNF-treated compared to saline-injected* T. cruzi*-infected mice. Bar represents the mean ± SD of the studied group (3 to 5 mice). These data represent three independent experiments.

At 150 dpi, all chronically infected C57BL/6 mice not-treated [25] or injected with saline present increased FN deposition in the heart tissue compared to NI controls (Figure 8). More importantly, anti-TNF therapy significantly reduced the frequency of areas positively stained for FN deposition in the cardiac tissue (Figure 8).

## 4. Discussion

Here we used the immunomodulatory strategy with the anti-TNF Infliximab antibody to investigate the influence of TNF on unbalanced immune response and cardiac tissue damage in chronic experimental Chagas' heart disease. TNF blocking therapy did not interfere with* T. cruzi* growth control but reshaped the broad immunological unbalance associated with CCC severity. Anti-TNF therapy selectively downregulated inflammatory cytokines but favored the expression of IL-10. Further, TNF neutralization reshaped parasite-specific CD8^+^ T-cells as CTL activity was reduced but IFN*γ* production preserved. Additionally, Infliximab remodeled chronic myocarditis as the number of Pfn^+^ cells was reduced but IFN*γ* ^+^ cells were unaffected. Moreover, therapy with Infliximab ameliorated ECG abnormalities and cardiac tissue damage. Therefore, we highlighted the contribution of TNF signaling as a hub in the immunological unbalance associated with the cardiac form of chronic* T. cruzi* infection.

When C57BL/6 mice were infected with low inoculum of the Colombian* T. cruzi* strain, 80–85% of mice survived the acute phase and developed CCC featured by low-grade cardiac tissue parasitism and CD8-enriched inflammation [18, 26]. Moreover, this model of chronic CD recapitulated ECG abnormalities and cardiac tissue injuries [18] found in chagasic patients [6] and non-human primates [23]. Similarly to CD patients [1–5], high TNF levels are detected in the serum of chronically Colombian-infected C57BL/6 mice paralleling CCC [18, 21, 25]. Therefore, this was an appropriated model to approach TNF influence on CCC pathogenesis.

Previous data showed cachexia and death in acutely* T. cruzi*-infected mice injected with the anti-TNF TN3 antibody [27] and remarkable body weight loss in chronically infected hamsters treated with the TNF blocker Etanercept [15]. Here, we showed that anti-TNF therapy with Infliximab was not toxic to chronically infected mice, as they survived and did not lose body weight. Importantly, Infliximab administration to chronically infected C57BL/6 mice significantly reduced* T. cruzi*-induced splenomegaly, a cue previously associated with* T. cruzi*-induced T- and B-cell polyclonal activation [28]; therefore, anti-TNF therapy interfered with an immunological hallmark of* T. cruzi* infection.

The low-grade CD8-enriched CCC [29] is reproduced in Colombian-infected C57BL/6 mice [18, 26]. Anti-TNF therapy was shown to reduce the CD8-enriched myocarditis in acute* T. cruzi* infection in mouse [12] and chronic infection in rats [16]. Corroborating these findings, Infliximab administration also reduced the number of CD8^+^ cells infiltrating the cardiac tissue of Colombian-infected C57BL/6 mice. Considering that part of the CD8^+^ cells infiltrating the cardiac tissue play a role in the control of* T. cruzi* growth [18], the anti-TNF-induced reduction of myocarditis was expected to have a deleterious result on* T. cruzi* parasitism. However, anti-TNF therapy did not abrogate parasite control systemically or in the cardiac tissue of chronically infected C57BL/6 mice, reinforcing previous study [16].

TNF mRNA expression is related to protein production in the cardiac tissue in experimental CCC [24, 30]; therefore, increased TNF mRNA expression in the heart tissue of Colombian-infected C57BL/6 mice supports that this model mimics the TNF-enriched milieu of patients with CCC [7, 8]. Importantly, blockade of TNF activity by Infliximab directly or indirectly reverberated in TNF mRNA expression. Although significantly reduced in Infliximab-treated mice compared to saline-injected ones, TNF mRNA expression was not abolished in the heart tissue. Hence, after the initial trigger by* T. cruzi* infection the elevated local TNF production may contribute to maintain high TNF mRNA expression in the cardiac tissue. The source of TNF mRNA expression in the cardiac tissue is unclear; however, both myocardial [31] and heart infiltrating inflammatory cells [7] may contribute to amplify local TNF production in chagasic infection. Notably, as TNF expression is not abolished by anti-TNF therapy, the remnant cytokine may contribute to parasite growth control [13, 31], explaining the absence of parasite burden after immunotherapy with Infliximab.

Along with the increased plasma TNF levels, we showed that chronically infected C57BL/6 mice present a broader systemic inflammatory profile with increased levels of IFN*γ*, IL-10, IL17A, IL-6, and NO_*x*_ in the serum, reproducing features of cardiopathic CD patients [1–5]. Although anti-TNF therapy reduced the circulating TNF and IFN*γ* levels, the production of these cytokines was not abolished. Moreover, the anti-TNF effect on cytokine production was selective as the high plasma IL-10 levels persisted and, consequently, after TNF blocking therapy the IL-10/TNF and IL-10/IFN*γ* ratios increased. These findings emphasize that the effectiveness of immune mediators involved in* T. cruzi* control [32] is disconnected from the systemic inflammatory profile [4, 5] and preserved after anti-TNF therapy. Indeed, no reactivation of* T. cruzi* in the heart and, mostly, in the central nervous system, which depends on preservation of the IFN*γ* axis [33], was detected after anti-TNF therapy.

TNF signals via TNFR1 and TNFR2 and TNFR1 signaling mediate most of the biological activities of TNF [34]. Due to TNFR1 upregulation on T-cells in chronically infected mice, we explored the effect of anti-TNF on TNFR1 expression. Indeed, TNF blockage abrogated* T. cruzi*-triggered TNFR1 upregulation on splenic CD4^+^ and CD8^+^ T-cells. Although not tested, it is reasonable to expect a similar effect on other cell types, including cardiomyocytes. TNFR1, but not TNFR2, signaling participates in* T. cruzi* control during acute infection [13, 35] and in acute heart injury [12]. Our data support that TNFR1 signaling is not crucial for parasite control during chronic infection. Importantly, TNF by acting on TNFR1, but not TNFR2, aggravates noninfectious heart failure [36, 37]. Therefore, TNFR1 downmodulation may be a favorable effect of anti-TNF on Chagas' heart disease. It is feasible that the beneficial effects of the anti-TNF therapy are due to an intervention in the positive feedback circuit triggered by an increase in TNF induced by* T. cruzi* infection, which may promote TNFR1 expression and the TNF/TNFR1 signaling that fuels TNF overproduction. Therefore, the downregulatory effect of anti-TNF on TNFR1 expression may contribute to reduce the responsiveness of activated cells to TNF in chronic chagasic infection and, therefore, disrupting or, at least, quelling the continuous TNF/TNFR1 signaling.

In chronic* T. cruzi* infection, TNF production by innate (Ly6C^+^ and F4/80^+^) immune cells was significantly enhanced. Interestingly, TNF production by Ly6C^+^ and F4/80^+^ monocytic cells was significantly reduced after anti-TNF therapy. Moreover, TNF neutralization increased the frequencies of IL-10^+^ and TNF^+^IL-10^+^ multifunctional producers among Ly6C^+^ and F4/80^+^ splenic cells. Again, the IL-10/TNF ratio was favored by Infliximab administration in the chronic phase of chagasic infection. However, it remains to be explored whether anti-TNF therapy alters the balance between classically activated inflammatory (M1) and alternatively activated regulatory (M2) macrophages in chronic* T. cruzi* infection, as recently shown in* Leishmania* infection [38]. Importantly, the study of chronic patients with polar clinical forms of CD showed that monocytes from IND patients display regulatory profile with high IL-10 production, whereas monocytes from cardiopathic patients may be committed to induction of inflammatory responses related to high TNF expression [17]. Further, the development of CCC in a canine model was related to high IFN*γ* and TNF and low IL-10 production systemically and in the heart tissue in the acute* T. cruzi* infection [39]. Therefore, our data support that the fate of the chronic Chagas' heart disease may be altered even after it had apparently been sealed.

Adaptive immune cells, particularly CD4^+^ and CD8^+^ T-cells, contribute to the inflammatory/regulatory cytokine balance in chronic CD [40]. In chronically* T. cruzi*-infected C57BL/6 mice the short anti-TNF therapy did not impact the increased TNF expression by T-cells. Increased frequencies of IL-17A^+^ and IL-10^+^ splenic CD4^+^ T-cells were also detected in chronically infected mice. The anti-TNF therapy downregulated the frequency of Th17 cells in these mice, corroborating previous findings in ankylosing spondylitis patients [20]; therefore, downmodulation of IL-17 is a conserved effect of TNF blocking. IL-17A contributes to protection against bacterial and fungal infections [41, 42]. In acute* T. cruzi* infection, IL-17 shaping the Th1 differentiation, cytokine and chemokine production, regulates the influx of inflammatory cells to the cardiac tissue involved in parasite resistance and myocardial destruction [43]. Here we demonstrated that high IL-17A production in the chronic phase of infection is not essential to anti-*T. cruzi* immunity. Importantly, IL-17A amplifies TNF signals by promoting mRNA stabilization of TNF-induced genes as chemokines and cytokines [44]; therefore, in breaking this circuit the beneficial effect of anti-TNF reducing the systemic inflammatory profile in chronic* T. cruzi* infection may reside. Nevertheless, anti-TNF therapy increased the frequency of IL-10-producing splenic CD4^+^ T-cells, favoring a more regulatory balance. Actually, IL-10-production by T-cells promotes* T. cruzi* control and protection from fatal acute myocarditis [45] and, thus, may concur to the beneficial effects of anti-TNF in chronic infection. More importantly, higher IL-10 expression was associated with better left ventricular ejection fraction and left ventricular diastolic diameter values in CD patients [5]. Our data support that anti-TNF therapy alters the inflammatory/regulatory balance in innate and adaptive immune effectors during chronic* T. cruzi* infection. Interestingly, considering the overall cytokine profile of innate and adaptive immune cells from CD patients, 80% of CCC patients are high inflammatory (e.g., IFN*γ*, TNF) cytokine producers and 75% of IND patients are high regulatory (e.g., IL-10) cytokine producers [40]. Therefore, our data support that the blend of inflammatory/regulatory cytokines in different sources and compartments of the host may contribute to the generation but also to the perpetuation of pathological features of chronic Chagas' heart disease, as previously hypothesized [46]. Moreover, our data support that the inflammatory scenario can be reshaped by appropriate therapeutic intervention.

The complete abrogation of TNF production or activity may not be required for the beneficial effect of TNF-based therapy on CCC and may even be harmful in* T. cruzi* infection. Nonbeneficial effects of anti-TNF immunotherapy in chronic inflammatory diseases (as rheumatoid arthritis and ankylosing spondylitis) associated with reactivation of* Mycobacterium* infection may be attributed to the depletion of the cytolytic CD8^+^perforin^+^/granulysin^+^ CTL population by Infliximab [19]. In chronically* T. cruzi*-infected mice treated with anti-TNF, the frequency of H2-K^b^-restricted VNHRFTLV-specific CTL activity effectors was decreased but IFN*γ* producers were preserved. In* T. cruzi* infection, CD8^+^ IFN*γ* ^+^ cells are protective and, conversely, CD8^+^Pfn^+^ cells are detrimental to electrical conduction and cardiac tissue injury [18]. Moreover, anti-TNF therapy in the chronic infection reduced the number of Pfn^+^ but preserved IFN*γ* ^+^ cells invading the heart tissue. Thus, the remolded immune response in the cardiac tissue may contribute to avoid parasite burden and, moreover, may improve electrical conduction, as we previously hypothesized [18].

To our knowledge, this is the first description of a beneficial effect of anti-TNF therapy on major immunological features previously shown to parallel CCC [1–5]. In* T. cruzi* infection, anti-TNF therapy reduced acute cardiomyocyte lesion [12] and reversed chronic depressive-like behavior [21]. Here, we demonstrated that in chronically* T. cruzi*-infected C57BL/6 mice, anti-TNF ameliorated important signs of Chagas' heart disease, including bradycardia, prolonged P wave duration and PR interval, ART and AVB2, and remarkable CD features [6]. In CD, the severity of heart failure parallels plasma TNF levels [1–5]. Recently, we showed that in murine models of severe and mild CCC disease severity paralleled TNF and NO levels in the serum [25]. Also, in chagasic patients the severity of myocardial scarring, assessed by magnetic resonance, was related to ECG QRS score [47]. Importantly, the prolonged QRS in Colombian-strain C57BL/6 infected mice was reduced by anti-TNF therapy paralleling the inhibition of FN overdeposition in the heart tissue. Lastly, enhanced FN deposition in the cardiac tissue reveals fibrosis in CD [48, 49]. These data reinforce the idea that* T. cruzi*-induced cardiac fibrosis can be ameliorated if the trigger of the process subsides [23] and posed TNF as a pivotal player in cardiac tissue injury and electrical abnormalities, supporting an even broader action of TNF signaling in chronic* T. cruzi* infection.

There is a consensus that* T. cruzi* persistence and parasite-driven deregulation of the immune response are key players in the establishment of CCC [29]. However, neither the intensity of parasitism nor the intensity of inflammation in the cardiac tissue seems to be crucial factors concurring to severity of chronic Chagas' heart disease. As hypothesized [18], the presence of Pfn^+^ cells in the cardiac inflammation, probably fuelled by TNF, is a key player in CCC pathogenesis. Furthermore, TNF itself may trigger and/or maintain the nonbeneficial systemic inflammatory profile and the detrimental cardiac inflammation, which may crucially contribute to the pathogenesis of the heart disease in chronic* T. cruzi* infection, supporting that these are interconnected events.

## 5. Conclusions

The short anti-TNF therapeutic scheme used was safe and prevented progression of and, moreover, reversed heart injury and ECG abnormalities in association with the reshape of immunological unbalance. Further, TNF blocking inhibits the harmful immune circuits involved in heart injury but preserves the beneficial antiparasitic immunity. Therefore, anti-TNF might be a viable treatment for chronic Chagas' heart disease combined with a trypanocidal drug. Although the molecular mechanisms linking TNF to major* T. cruzi*-induced immunological abnormalities remain to be clarified, our data open a new pathway to be explored to comprehend the pathogenesis of Chagas' heart disease.

## Supplementary Material

Supplementary Figure S1: The high levels of IL-17A, IL-6 and NO_x_ were detected in the serum of chronically *T. cruzi*-infected mice (Figures S1(a) and S1(b)), resembling chronic chagasic patients [4]. Anti-TNF therapy tended to decrease the levels of IL-17A, Il-6 and NO_x_ in the serum in comparison with saline-injected chronically infected mice.
Supplementary Figure S2: At 120 dpi, chronically Colombian-infected C57BL/6 mice show CD8-enriched myocarditis [18]. Anti-TNF therapy did not diminish the general inflammatory foci composed of CD8+, CD4+, and F4/80+ macrophages) (Figure S2(a)) but reduced the number of CD8+ cells infiltrating the cardiac tissue (Figures S2(b) and S2(c)). Therefore, we tested whether it was a site restricted effect examining the cell composition of a secondary lymphoid tissue. The frequencies of CD4+ and CD8+ T-cells in the spleen persisted unchanged after anti-TNF administration (Figure S2(d)).
