# Supplementary material for: Tumor Necrosis Factor Is a Therapeutic Target for Immunological Unbalance and Cardiac Abnormalities in Chronic Experimental Chagas' Heart Disease
Source: Mediators Inflamm. 2014 Jul 22;2014:798078. doi: 10.1155/2014/798078 (PMC4130030; doi:10.1155/2014/798078)
Supplement: Supplementary file 1 — Supplementary Figure S1: The high levels of IL-17A, IL-6 and NOx were detected in the serum of chronically T. cruzi-infected mice (Figures S1(a) and S1(b)), resembling chronic chagasic patients [4]. Anti-TNF therapy tended to decrease the levels of IL-17A, Il-6 and NOx in the serum in comparison with saline-injected chronically infected mice. Supplementary Figure S2: At 120 dpi, chronically Colombian-infected C57BL/6 mice show CD8-enriched myocarditis [18]. Anti-TNF therapy did not diminish the general inflammatory foci composed of CD8+, CD4+, and F4/80+ macrophages) (Figure S2(a)) but reduced the number of CD8+ cells infiltrating the cardiac tissue (Figures S2(b) and S2(c)). Therefore, we tested whether it was a site restricted effect examining the cell composition of a secondary lymphoid tissue. The frequencies of CD4+ and CD8+ T-cells in the spleen persisted unchanged after anti-TNF administration (Figure S2(d)). [file 798078.f1.pdf]

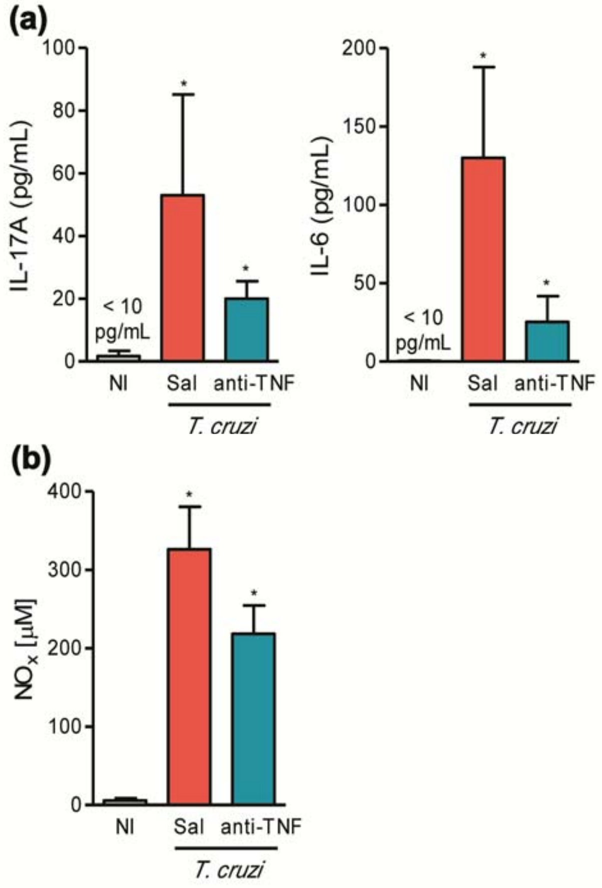

FIGURE S1. Effects of therapy with Infliximab on IL-17A, IL-6 and NO_x concentrations in the serum of chronically *Trypanosoma cruzi*-infected mice. C57BL/6 mice infected with 100 bt of the Colombian *T. cruzi* strain were treated from 120 to 150 dpi and analyzed at 150 dpi. (a) Measure of IL-17A and IL-6 concentrations in the serum. (b) Concentration of NO_x (nitrite/nitrate) levels in the serum. $^{*}P < 0.05$, *T. cruzi*-infected mice (saline-injected or anti-TNF-treated) compared to NI controls. Bar represents the mean ± SD of the studied group (3 to 5 mice). These data represent three independent experiments.

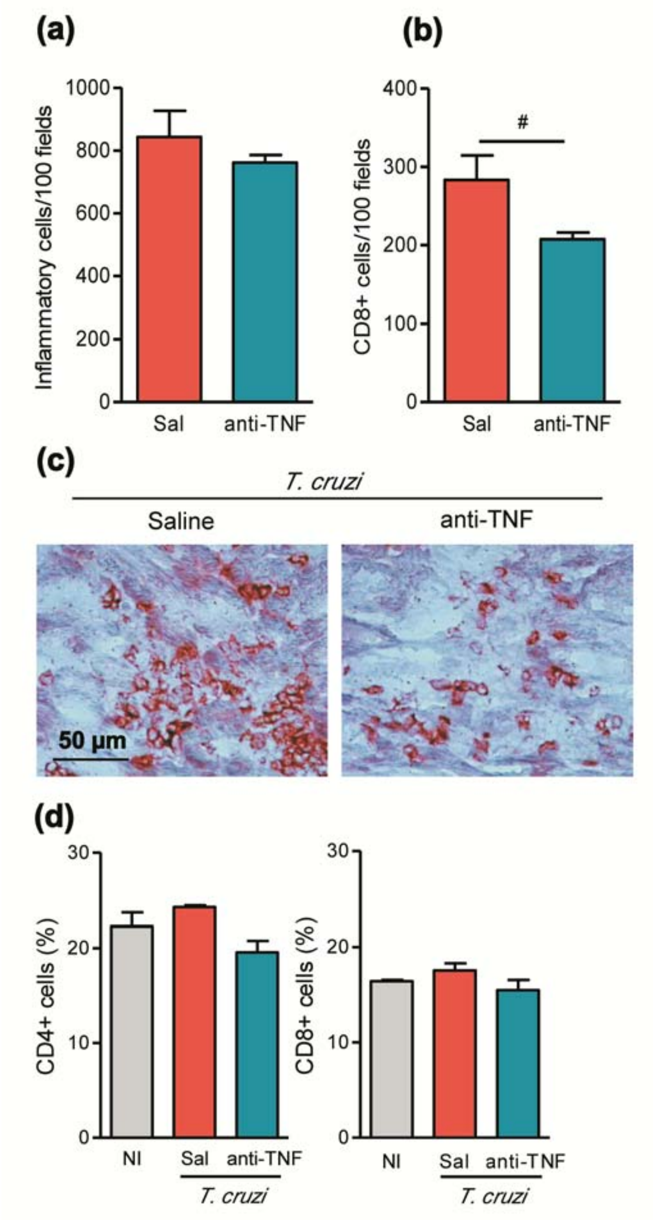

FIGURE S2. Anti-TNF therapy reduces the number of $CD8^+$ cells infiltrating the cardiac tissue of chronically *Trypanosoma cruzi*-infected mice. (a) Total inflammatory cells invading the heart tissue. (b) Number of $CD8^+$ cells infiltrating the cardiac tissue. (c) Representative heart sections from each experimental group analyzed by immunohistochemical staining to detect $CD8^+$ cells. (d) Frequency of $CD4^+$ and $CD8^+$ T-cells in the spleen [R1 (SSCxFSC) gated]. $^{\#}P < 0.05$, anti-TNF-treated compared to saline-injected *T. cruzi*-infected mice. Bar represents the mean ± SD of the studied group (3 to 6 mice). These data represent three independent experiments.
